# miR-588 is a prognostic marker in gastric cancer

**DOI:** 10.18632/aging.202212

**Published:** 2020-12-15

**Authors:** Yongyi Chen, Jie Zhang, Wangang Gong, Wumin Dai, Xiaohong Xu, Songxiao Xu

**Affiliations:** 1 The Cancer Hospital of the University of Chinese Academy of Sciences, Zhejiang Cancer Hospital, Hangzhou, China; 2 Institute of Basic Medicine and Cancer (IBMC), Hangzhou, China; 3 Chinese Academy of Sciences, Hangzhou, Zhejiang, China; 4 Zhejiang Provincial Center for Drug and Medical Device Procurement, Hangzhou, China

**Keywords:** gastric cancer, miR-588, immune infiltration, bioinformatics analysis

## Abstract

In an effort to identify a novel microRNA (miRNA) as a gastric cancer (GC) treatment target and prognostic biomarker, we surveyed The Cancer Genome Atlas database and found that miR-588 expression is low in GC tissues. This was confirmed by real-time reverse transcription polymerase chain reaction assays of GC patient plasma samples and SGC7901 and MNK28 cells. A constructed miRNA-mRNA network showed that CXCL5, CXCL9, and CXCL10 are target genes of miR-588. Analysis of the miRWalk database revealed that miR-588 directly binds to CXCL5 and CXCL9. Overexpression of miR-588 reduced GC cell proliferation *in vitro* and *in vivo*. High expression of miR-588 inhibited Ki-67 expression *in vivo*. The FunRich database also showed that CXCL5, CXCL9, and CXCL10 are involved in immune responses, while the Database of Immune Cell Expression showed they are differentially expressed in CD8+ T cells. High expression of CXCL9 and CXCL10 correlated positively with infiltrating levels of CD4+ T and CD8+ T cells in stomach adenocarcinoma. High expression of miR-588, CXCL5, CXCL9, and CXCL10 was associated with prolonged survival of GC patients. These findings indicate that miR-588 is a biomarker for tumor-associated immune infiltration and a prognostic marker in GC patients.

## INTRODUCTION

Gastric cancer (GC) is a worldwide public health issue and the second leading cause of cancer death. GC is caused by various factors, including genetic and environmental factors [1–3]. Although advances have been made in treatment strategies, the survival rate for GC patients is still unsatisfactory, with a 5-year survival rate of only 20% to 40% [4]. There is an urgent need to find novel biomarkers of GC and elucidate the molecular mechanisms of GC pathogenesis to improve patient outcomes.

MicroRNAs (miRNAs) are a class of single-stranded, noncoding RNAs that consist of 19 to 25 nucleotides [5]. Many miRNAs have been reported to affect human pathologic and physiologic processes through a range of molecular mechanisms [6, 7]. Furthermore, aberrant expression of miRNAs leads to a variety of diseases, including cancer, by interacting with the 3′-untranslated region of target mRNAs and altering protein translation [8]. Increasing evidence shows that miRNAs are involved in many biologic processes, including proliferation, apoptosis, migration, and differentiation, which suggests that miRNAs have the potential to be promising targets for cancer treatment [9, 10].

Recently, miR-588 was reported to be dysregulated in multiple human cancers, including lung, ovarian, and prostate cancers. In lung cancer, miR-588 suppressed tumor cell migration and invasion and was also significantly associated with advanced lung squamous cell carcinoma [11]. In ovarian cancer, miR-588 was overexpressed and associated with ovarian cancer risk alleles [12]. However, limited studies have investigated the function of miR-588 in GC.

In this study, we analyzed three GC miRNA microarray data sets as well as serum of GC patients and found low expression of miR-588 in GC. As predicated by bioinformatics, miR-588 directly targeted CXC chemokine ligand 5 (CXCL5), CXCL9, and CXCL10, which are associated with tumor-infiltrating immune cells in GC.

## RESULTS

### miR-588 expression was low in patients with GC

**Figure 1:**
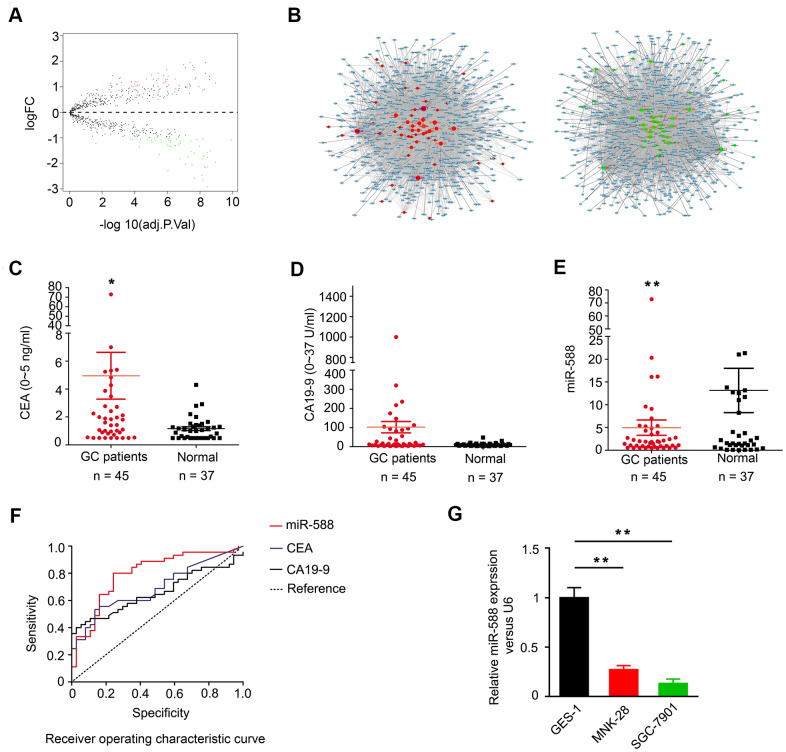
**Expression of miR-588 in TCGA database and serum of GC patients.** (**A**) Volcano map of the differential expression of miRNAs in gastric cancer. (**B**) The miRNA-mRNA network; green diamonds are downregulated miRNAs. (**C**, **D**) Expression of CEA and CA 19-9 was higher in GC (n = 45) compared to normal samples (n = 37). (**E**) miR-588 expression was low in serum of GC patients. (**F**) Receiver operating characteristic (ROC) curves of miR-588, CEA, and CA 19-9. (**G**) RT-PCR analysis of miR-588 expression relative to control U6+ expression in GES-1, MNK28, and SGC7901 cells.

Gene Expression Omnibus (GEO) database was analyzed to determine the expression pattern of miRNAs in GC patients. As shown in the volcano map in Figure 1A, 60 upregulated miRNAs and 54 downregulated miRNAs were identified in GC (Supplementary Tables 1, 2). Analysis of the TargetScan database identified 54 downregulated miRNAs; miR-588 was shown to regulate a total of 3,728 genes by axes, including CXCL5, CXCL9, and CXCL10 (Figure 1B). We next analyzed serum levels of miR-588 and two GC markers, carcinoembryonic antigen (CEA) and carbohydrate antigen 19-9 (CA 19-9), in GC patients and healthy individuals. We found higher serum levels of CEA and CA 19-9 and lower miR-588 levels in GC patients compared with healthy individuals (Figure 1C–1E and Table 1). We further compared the areas under the curve for miR-588, CEA, and CA 19-9 and found that miR-588 had a higher sensitivity and specificity (Figure 1F). We examined miR-588 expression in several GC cell lines, including GES-1, MNK28, and SGC7901, and found miR-588 expression to be highest in GES-1 (Figure 1G).

**Table 1:** Clinical and tumor marker characteristics in gastric cancer patients and controls.

**Groups**	**Age, (years)**	**Male, n (%)**	**miR-588, (P_25_,P_75_)**	**CEA, (P_25_,P_75_)**	**CA199, (P_25_,P_75_)**
Gsatric (n = 45)	58.55±8.86	29 (64.44%)	0.20 (0.05,0.88)**	1.84 (0.85,4.56)**	14.99 (7.83,99.45)*
Controls (n = 37)	42.43±11.27	22 (59.45%)	2.13 (0.85,12.64)	1 (0.5,1.41)	9.52 (5.94,14.67)

* Compared with controls: *p* < 0.05. **Compared with controls: *p* < 0.01.

### miR-588 overexpression reduced the viability of GC cells by increasing CXCL5, CXCL9, and CXCL10 expression

**Figure 2:**
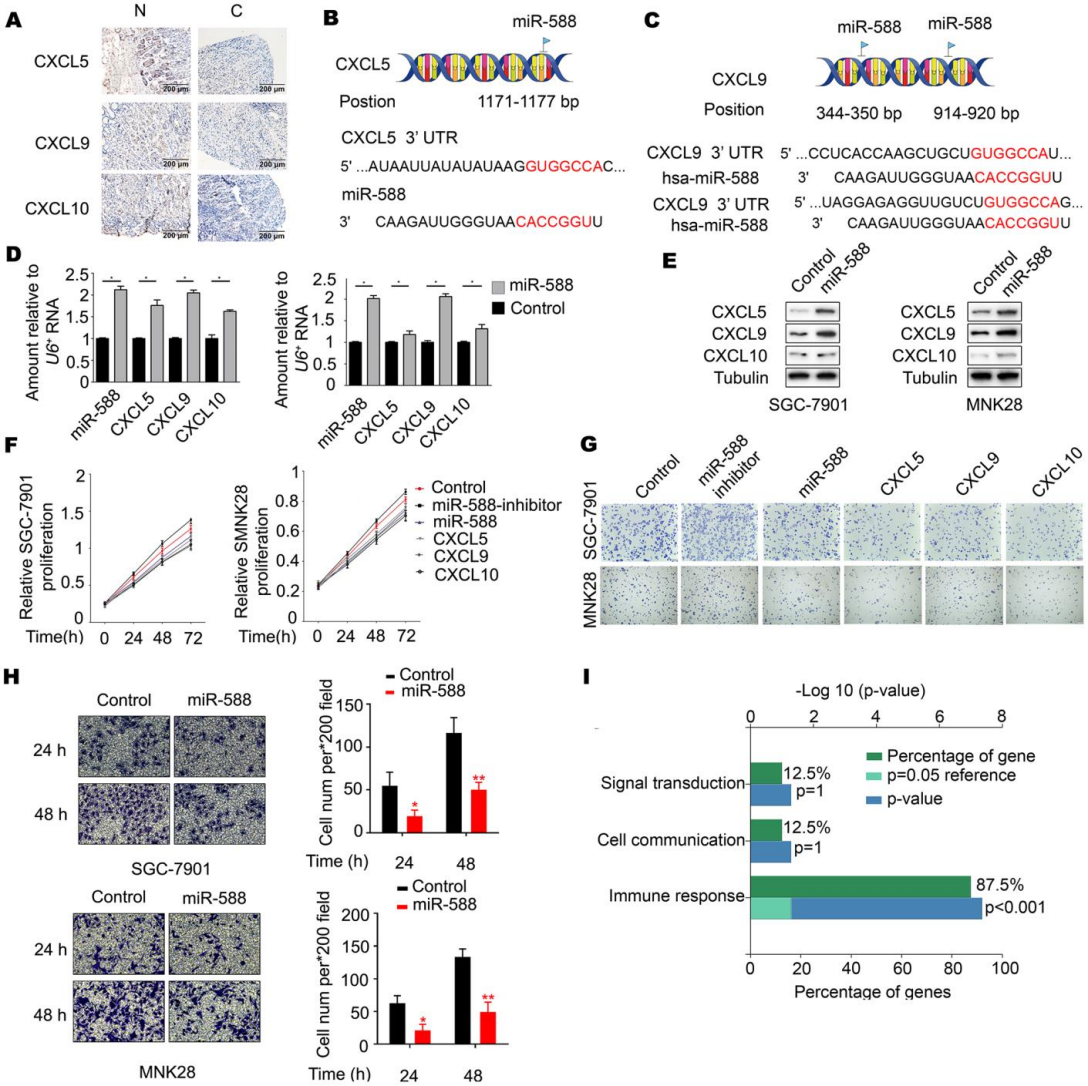
**miR-588 as a novel regulator of CXCL5, CXCL9, and CXCL10 expression.** (**A**) Immunohistochemistry of CXCL5, CXCL9, and CXCL10 expression in gastric tumors and corresponding nontumor adjacent gastric tissues. (**B, C**) Possible binding sites between miR-588 and CXCL5 and between miR-588 and CXCL9. (**D**) RT-PCR analysis of CXCL5, CXCL9, and CXCL10 mRNA relative to a control U6+. The relative level in MNK28 and SGC7901 cells was arbitrarily designated as 1. Each column represents the mean±SD from three biologic repeats. (**E**) Expression of CXCL5, CXCL9, and CXCL10 was examined using western blot analysis after overexpression of miR-588. (**F**) The cell viability inhibitory rate was determined using an MTT assay. MNK28 and SGC7901 cells were transfected with GV268-miR-588. (**G**) Crystal violet assay was performed to test cell growth after 48 hours of increased expression of miR-588, CXCL5, CXCL9, and CXCL10 in gastric cancer cells. (**H**) Metastatic capacity of MNK28 and SGC-7901 cells after 24 and 48 hours of increased expression of miR-588. (**I**) CXCL5, CXCL9, and CXCL10 focus on the immune response.

To determine the association between miR-588 and CXCL5, CXCL9, and CXCL10, levels of CXCL5, CXCL9, and CXCL10 were measured in GC tumor tissues and corresponding nontumor adjacent gastric tissues. Immunohistochemistry staining showed abundant and uniform expression of CXCL5, CXCL9, and CXCL10 proteins in nontumor adjacent gastric tissues (N), but expression of these proteins was significantly downregulated in all tumor samples (C) (Figure 2A and Table 2). Analysis using the miRWalk database showed that miR-588 directly binds to CXCL5 at the 1171-1177 bp site and to CXCL9 at the 344-350 and 914-920 bp sites (Figures 2B, 2C). We next analyzed the interactions between CXCL5, CXCL9, and CXCL10 using STRING. As shown in Figure 2C, CXCL10 was found to interact with CXCL5 and CXCL9, which indicated that there may be an interaction between miR-588 and CXCL10. Real-time reverse transcription polymerase chain reaction (RT-PCR) analysis revealed significantly increased expression of CXCL5, CXCL9, and CXCL10 in MNK28 and SGC7901 cells after transfection with miR-588 mimics compared with mimic negative control (NC)-transfected cells (P < 0.05, Figure 2D). These results were consistent with changes in protein expression of CXCL5, CXCL9, and CXCL10 (Figure 2E). Moreover, proliferation of MNK28 and SGC7901 cells was significantly decreased after transfection with GV268-miR-588/CXCL5/CXCL9/CXCL10 (Figure 2F). In addition, crystal violet staining results were consistent with those of an MTT assay (Figure 2G; statistical analysis shown in Supplementary Figure 1). Overexpression of miR-588 significantly decreased the metastatic capacity of MNK28 and SGC7901 cells (Figure 2H). Remarkably, a functional enrichment analysis using the FunRich database showed that CXCL5, CXCL9, and CXCL10 function was mainly related to immune response (Figure 2I).

**Table 2:** Expression of CXCL5/9/10 in gastric cancer and normal tissue samples.

**Variables (n=30)**	**CXCL5 expression**		**CXCL9 expression**		**CXCL10 expression**	
	**Low**	**High**	**Low**	**High**	**Low**	**High**
Gastric cancer	26	4	25	5	24	6
Normal	3	27	2	28	5	25
P-value	<0.001		<0.001		<0.001	

Statistical analysis was performed with a ^a^χ^2^ test or ^b^Fisher’s exact test.

### CXCL5, CXCL9, and CXCL10 expression was high in CD8+ T cells

**Figure 3:**
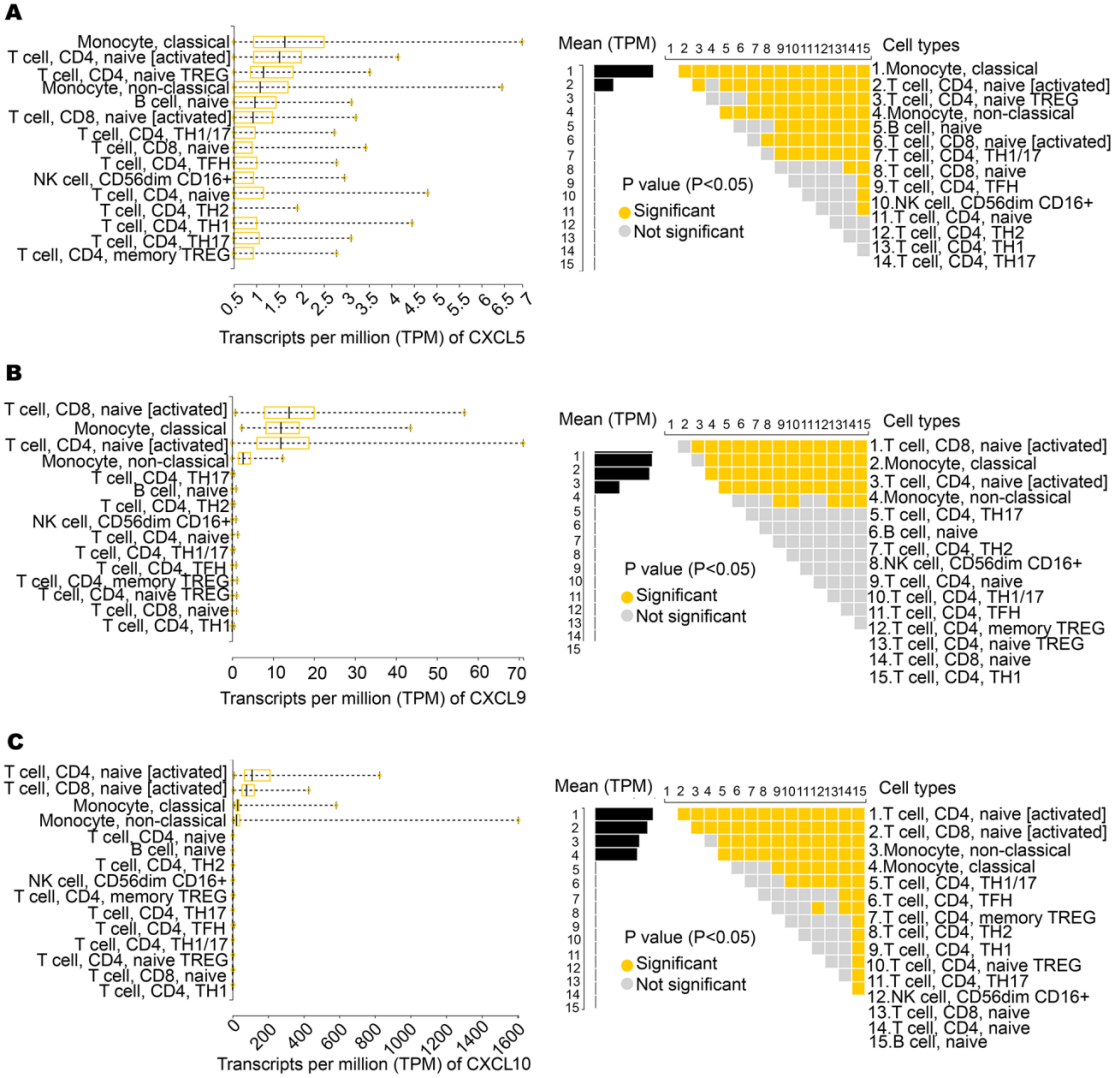
**Expression of CXCL5, CXCL9, and CXCL10 in immune cells.** (**A**) CXCL5 shows significantly high expression in monocytes, CD4^+^ T cells, and CD8^+^ T cells. (**B, C**) CXCL9 and CXCL10 show significantly high expression in CD4^+^ T cells and CD8^+^ T cells.

We further analyzed the expression of CXCL5, CXCL9, and CXCL10 in various immune cells. According to an analysis of the Database of Immune Cell Expression (DICE), CXCL5 is highly expressed in immune cells, including CD4+ and CD8+ T cells and monocytes (Figure 3A). As shown in Figures 3B, 3C, CXCL9 and CXCL10 were highly expressed in CD8+ and CD4+ T cells.

### CXCL5, CXCL9, and CXCL10 expression was correlated with immune infiltration in GC

**Figure 4:**
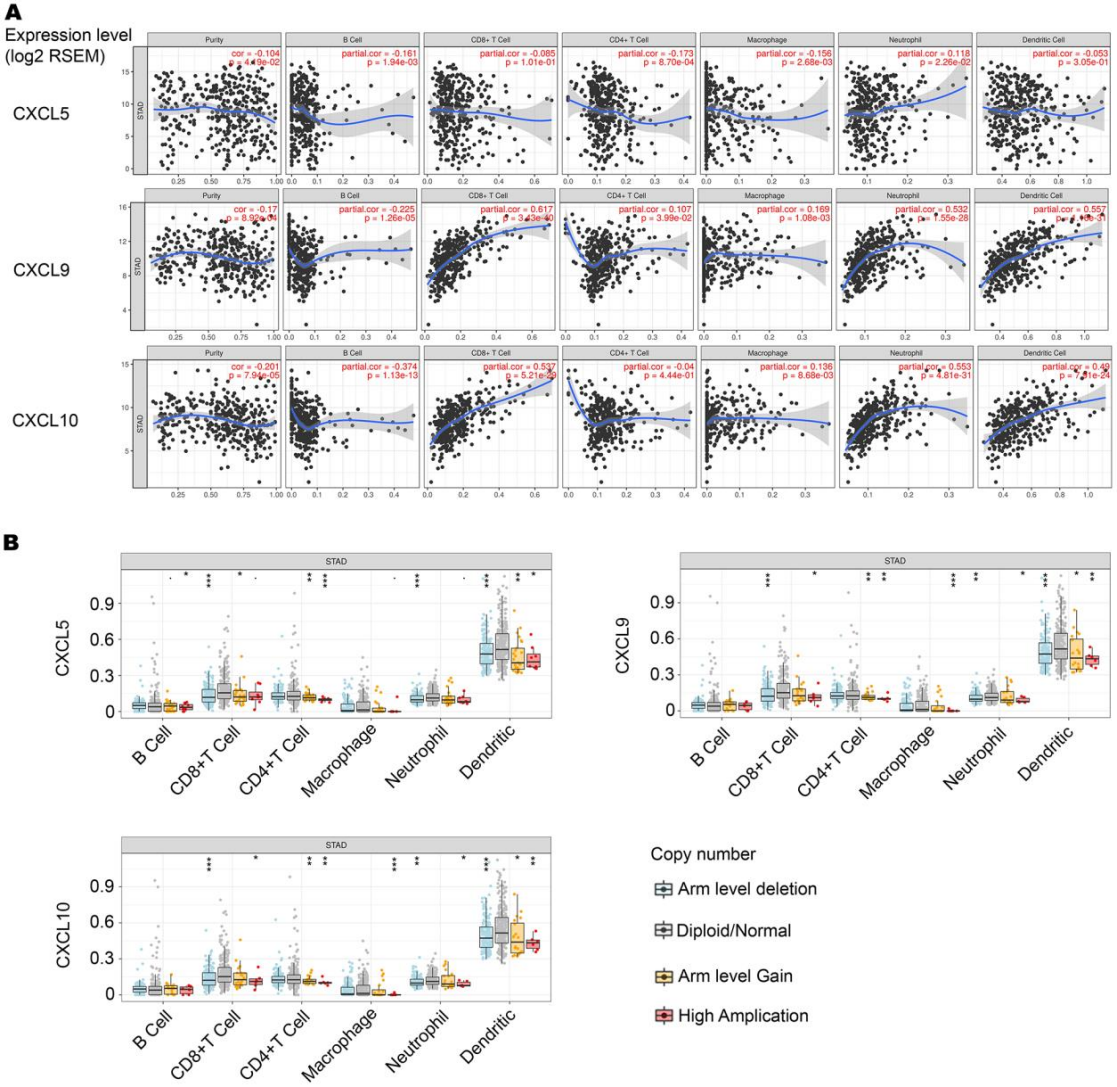
**Correlation between CXCL5, CXCL9, and CXCL10 expression and immune infiltration level in stomach adenocarcinoma (STAD).** (**A**) CXCL5 expression is negatively correlated with tumor purity, and this correlation is significant. CXCL5 expression is not significantly correlated with infiltrating levels of CD8^+^ T cells or CD4^+^ T cells (n = 415). CXCL9 and CXCL10 expression is negatively correlated with tumor purity, and this correlation is significant. CXCL9 and CXCL10 expression is positively correlated with infiltrating levels of CD8^+^ T cells and CD4^+^ T cells (n = 415), and this correlation is significant. (**B**) Significant expression of normal CXCL5, CXCL9, and CXCL10 in CD8^+^ T cells.

A recent study showed that overexpression of CXCL5, CXCL9, and CXCL10 improved the immune function of cancer patients [13]. Therefore, we investigated whether CXCL5, CXCL9, and CXCL10 expression was correlated with immune infiltration levels in GC using the Tumor Immune Estimation Resource (TIMER) database. As shown in Figure 4A, expression of CXCL5, CXCL9, and CXCL10 was significantly negatively correlated with tumor purity. Expression of CXCL9 and CXCL10 was positively correlated with infiltrating levels of CD8+ T cells in stomach adenocarcinoma. Next, we analyzed the infiltration levels of different somatic copy number alterations for CXCL5, CXCL9, and CXCL10. We found diploid/normal levels of CXCL5, CXCL9, and CXCL10, despite high expression levels in CD8+ T cells (Figure 4B).

### Low miR-588 expression was significantly associated with increased GC cell proliferation *in vivo* and decreased overall survival in GC patients

**Figure 5:**
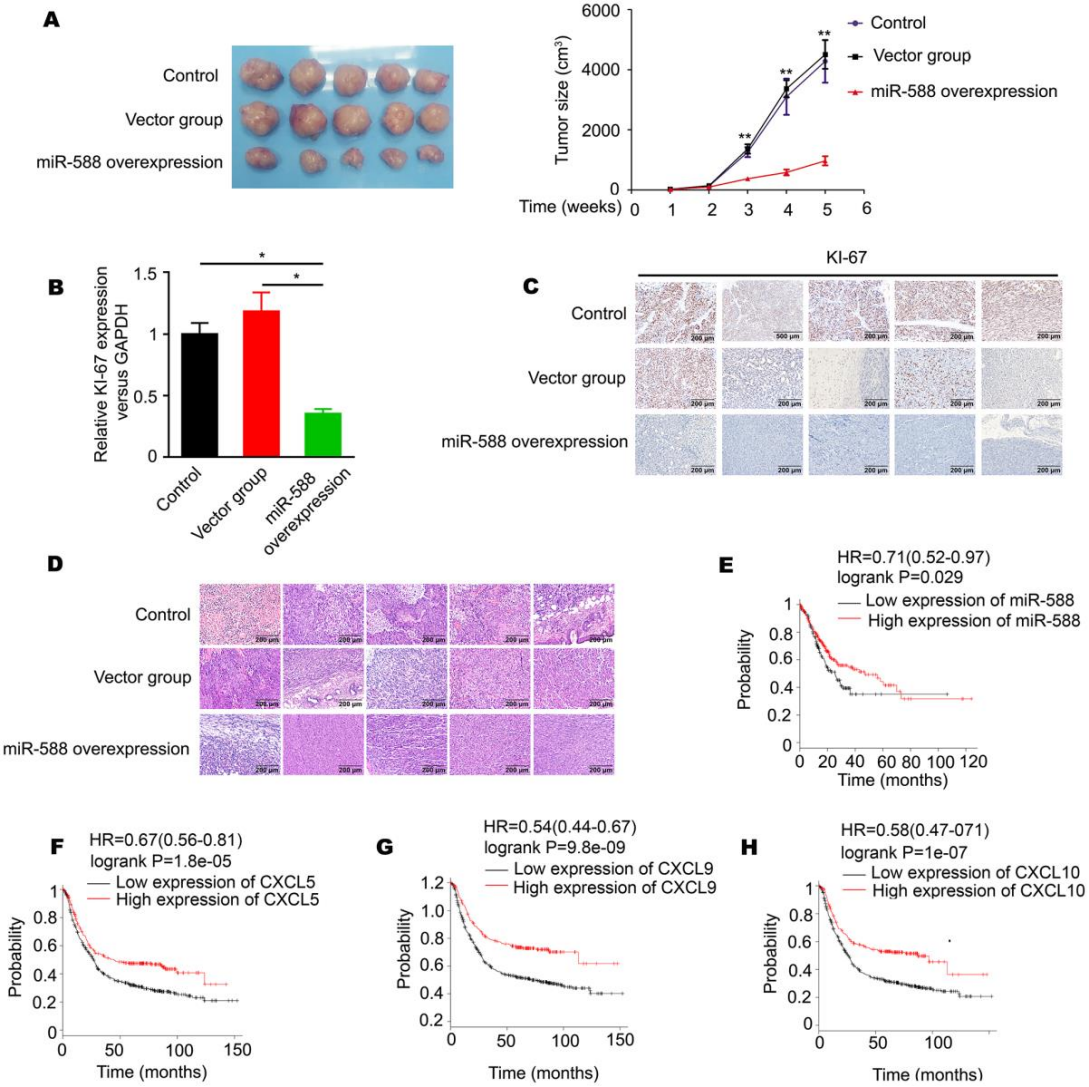
**Differential expression of miR-588 was significantly associated with GC cell proliferation *in vivo* and is a prognostic markers for GC patient survival (n = 876).** (**A**) High expression of miR-588 significantly inhibited GC cell proliferation *in vivo*. (**B**) High expression of miR-588 significantly downregulated Ki-67 expression in nude mice tumors. (**C**) Immunohistochemistry analysis of Ki-67 expression in control, vector, and miR-588 overexpression nude mice tumors tissues. (**D**) Hematoxylin and eosin staining was used for different groups of nude mice tumors. (**E**–**H**) High levels of miR-588, CXCL5, CXCL9, and CXCL10 were associated with prolonged survival of GC patients.

We investigated whether miR-588 was involved in proliferation of CG cells. Nude mice were divided into three groups: a control group, a vector group, and an miR-588 overexpression group; mice were injected subcutaneously (n = 5 per group). Tumor size was measured every 7 days after injection. Whole tumors were excised on the 35^th^ day. Resected tumors from the miR-588 overexpression group were significantly smaller than those from the control and vector groups (Figure 5A). RT-PCR analysis revealed significantly decreased expression of Ki-67 in the miR-588 overexpression group (P < 0.05, Figure 5B), which was consistent with Ki-67 protein expression determined using immunohistochemistry (Figure 5C). Furthermore, there was little distinction between the three groups after staining with hematoxylin-eosin (Figure 5D). To investigate whether miR-588, CXCL5, CXCL9, and CXCL10 are associated with prognosis in patients with GC, we mined the largest-scale survival-related clinical data sets from Kaplan-Meier Plotter (http://kmplot.com/). We found that high expression of miR-588, CXCL5, CXCL9, and CXCL10 was significantly associated with longer overall survival of GC patients (P < 0.05, Figures 5E–5H).

## DISCUSSION

miRNAs are well known to be involved in the development and progression of many cancers [13, 14]. Recently, miRNAs have been increasingly identified as important therapeutic targets in GC [15, 16]. In this study, we explored the expression, prognosis, and function of miR-588 in human GC. We found that expression of miR-588 was downregulated in GC patients when compared with healthy individuals. Overexpression of miR-588 decreased proliferation of GC cell lines SGC7901 and MNK28 and increased the expression of chemokines CXCL5, CXCL9, and CXCL10. We also demonstrated that miR-588 plays an important role in the development and progression of GC. More specifically, our data suggest that low expression of miR-588 is a poor prognostic factor in GC patients. Consistent with our findings, miR-588 has been reported to be expressed at low levels in lung squamous cell carcinoma and breast cancer, and this low expression was associated with unfavorable patient prognosis [11, 17]. In contrast, in human prostate cancer and ovarian cancer, miR-588 was shown to be overexpressed in tumor cells and tissues [14]. Thus, miR-588 expression level seems to have different functions in different pathologic conditions, which may be a result of tissue- or organ-specific differences.

The CXC chemokine family is a unique group of cytokines that are known for their distinct abilities in the regulation of angiogenesis [18]. CXCL5, a proangiogenic CXC-type chemokine, is also an inflammatory mediator and a powerful attractant for granulocytic immune cells [19]. Recently, studies have demonstrated that CXCL5 expression is correlated with tumor-derived angiogenesis, tumor growth, and metastasis [20, 21] and is also highly associated with inflammatory infiltrates [22]. CXCL9 and CXCL10 are angiostatic members of the CXC chemokine family that are induced by both type I interferons (IFN-α and IFN-β) and type II interferons (IFN-γ) [23]. CXCL9 and CXCL10 were reported to promote tumor suppression by increasing immune cell differentiation, migration, and activation [24]. Our study revealed that high expression of CXCL5, CXCL9, and CXCL10 prolonged GC patient survival. Moreover, we found that CXCL5, CXCL9, and CXCL10 were highly expressed in CD4+ and CD8+ T cells. CXCL9 and CXCL10 were positively correlated with CD8+ T cells. Previous studies have shown that CD4+ and CD8+ T cells provide protective immunity against tumors [25]. Furthermore, CD8+ T cells have been found to fight against intracellular pathogens and play a particularly important role in adaptive immunity of the host by producing effector cytokines, such as IFN-γ and TNF-α [26, 27]. Future experiments, particularly *in vivo* investigations, are required to elucidate the effects of miR-588 on tumor-associated immune infiltration in GC.

In conclusion, our study demonstrated for the first time that miR-588 upregulated CXCL5, CXCL9, and CXCL10 expression. This may play a critical role in promoting tumor-associated immune infiltration, thereby providing a novel potential approach for GC treatment.

## MATERIALS AND METHODS

### Exploration of the differentially expressed miRNAs

RNA sequencing (RNA-Seq) data were derived from the GEO database. There were 112 adjacent normal tissues and 412 gastric tumor tissues with available miRNA-Seq. We then used the R and Bioconductor package of edgeR to explore miRNAs that showed significantly different expression between normal and cancer tissues. Differentially expressed miRNAs and mRNAs are listed in Supplementary Tables 1, 2.

### Construction of the endogenous miRNA-mRNA network

The miRDB (http://www.mirdb.org/), TargetScan (http://www.targetscan.org/vert_71/), and miRanda (http://www.targetscan.org/vert/) databases were used to predict interactions between miRNAs and mRNAs. The Cytoscape software was used to construct a network of significantly differentially expressed miRNAs and mRNAs (miRNA-mRNA network), according to predicted interactions.

### Western blotting

MNK28 cells, SGC7901 cells, MNK28 cells transfected with GV268-miR-588, and SGC7901 cells transfected with GV268-miR-588 were harvested. For immunoblotting, cells were lysed by radioimmunoprecipitation assay (RIPA) buffer (P0013C, Beyotime) and centrifugated at 10,000 × g for 10 minutes. Extracted proteins were then resolved by sodium dodecyl sulfate–polyacrylamide gel electrophoresis (SDS-PAGE). Protein blots were incubated with appropriate antibodies, and protein bands were visualized by enhanced chemiluminescence (ECL) or ECLPLUS (Amersham, Piscataway, NJ). The antibodies used were CXCL5 (D263012-0025, BBI), CXCL9 (ab202961, Abcam), CXCL10 (D220389-0025, BBI), and antitubulin (#T8203, Sigma-Aldrich, St. Louis, MO). The GeneGnome HR system (Syngene, Cambridge, UK) was used to scan blots.

### Culture of SGC7901 and MNK28 cells

The SGC7901 and MNK28 GC cell lines were purchased from the China Center for Type Culture Collection (Wuhan, China). Cells were cultured in RPMI-1640 medium supplemented with 10% fetal bovine serum (FBS) and antibiotics (100 U/mL streptomycin and 100 U/mL penicillin) at 37° C in a humidified atmosphere containing 5% CO2.

### Real-time reverse transcription polymerase chain reaction (RT-PCR)

All RT-PCR processes were performed as previously described [28, 29]. The siRNA and specific miRNA, CXCL5, CXCL9, CXCL10, Ki-67, U6, and GAPDH primers were synthesized by Sangon Biotech (Shanghai, China), as listed in Supplementary Table 3.

### MTT assay

SGC7901 and MNK28 cells were seeded on 96-well plates at a density of 1 × 103 cells/well. Cells were incubated for 24 hours at 37° C and transfected with an miR-588 inhibitor (GUUCUAACCCAUUGUGGCCAA; Sangon Biotech, Shanghai Co., Ltd.), miR-588 mimics (Sense:UUGGCCACAAUGGGUUAGAAC; Antisense:UCUAACCCAUUGUGGCCAAUU; Sangon Biotech, Shanghai Co., Ltd.), GV268-CXCL5, GV268-CXCL9, or GV268-CXCL10 for 24, 48, 72, and 96 hours. Subsequently, 10 μL of MTT (5 mg/mL) was added to each well, and the cells were sequentially cultured at 37° C at 5% CO2 for 3 hours. Dimethyl sulfoxide (DMSO, 150 μL) was added to each well. Optical density was determined at 490 nm using a microplate reader (Bio-Rad Laboratories, Inc., Hercules, CA).

### Crystal violet assay

SGC7901 and MNK28 cells (1 × 10^5^ cells/well) were transfected with miR-588 mimics, GV268-CXCL5, GV268-CXCL9, and GV268-CXCL10 and were seeded in triplicate on six-well plates. Following 48 hours of culture at 37° C, culture medium was carefully removed. After cells were washed gently with 3 mL of warmed phosphate-buffered saline (PBS; P5368, Sigma-Aldrich), 750 μL of crystal violet solution (ECM101, Sigma-Aldrich) were added. Cells were incubated at room temperature for 10 minutes and then washed twice with tap water. Finally, the culture plate was allowed to drain upside down on paper towels before photographing.

### Tissue immunohistochemistry (IHC)

Primary GC tissue samples (n = 30) and corresponding nontumor adjacent gastric tissue samples (n = 30) were collected from the Biobank of Zhejiang Cancer Hospital. Prior to participating, each patient signed an informed consent sheet. This study was approved by the Medical Ethics Committee, Zhejiang Medical College. The immunohistochemical staining kit was purchased from Beijing Zhongshan Golden Bridge Biotechnology Co. Ltd. In brief, paraffin sections were successively deparaffinized, rehydrated, and boiled for antigen retrieval. Then, a primary CXCL5 (D263012-0025, BBI), CXCL9 (ab202961, Abcam), or CXCL10 (D220389-0025, BBI) (1:500) was added to each section for 2 hours at room temperature. After sections were gently washed three times in PBS, 400 μL of histochemical polymer enhancer were added for 20 minutes at room temperature. After gently washing three times in PBS, the secondary antibodies were added and incubated for 20 minutes, followed by washing, DAB staining, counterstaining, and mounting. The nude mice tissue immunohistochemistry was also performed in this way, and Ki-67 (YM1407, Immunoway) (1:200) was added to each section.

### Hematoxylin-eosin (HE) staining

Nude mice were divided into three groups: a control group, a vector group, and a miR-588 overexpression group. The tumor tissue of those groups was used for HE staining. The HE staining kit was bought from Beyotime. Procedures were followed according to the HE staining kit instructions (Beyotime, C0105).

### Animal experiments

Six-week-old nude mice (n = 15) were purchased from SLAC Laboratory Animal Co., Ltd. (Shanghai, China) and were randomly divided into three groups. SGC7901 cells (1 × 10^7^ cells/mL, control group) infected with lentivirus vector (1 × 10^7^ cells/mL, vector group) and SGC7901 cells infected with miR-588 lentivirus (1 × 10^7^ cells/mL, overexpression group) were injected subcutaneously on the back of each nude mouse (0.2 mL). Average tumor volume was measured three times per week. At the termination of the experiment (on the 35^th^ day), mice were sacrificed and tumors were excised for volume measurements.

### Functional enrichment analysis

Functional enrichment of CXCL5, CXCL9, and CXCL10 was analyzed using FunRich (http://www.funrich.org/). FunRich is a stand-alone software tool for functional enrichment and interaction network analysis of genes and proteins. The analysis results can be depicted graphically using Venn, bar, column, pie, and doughnut charts. The FunRich database currently supports the enrichment analysis of the following categories: biologic processes, cellular components, molecular functions, protein domains, biologic pathways, transcription factors, and clinical synopsis phenotypic terms.

### Upregulation of miR-588 GC stable strains

The miR-588 upregulation lentivirus was constructed by GeneChem (Shanghai GeneChem Co., Ltd.). MNK28 and SGC7901 cells were seeded in six-well plates (2 × 10^5^ cells/well) and were cultured in RPMI-1640 medium supplemented with 10% FBS at 37° C for 16 to 24 hours. Then, 20 μL of miR-588 lentivirus and 3 μL of polybrene (Solarbio, H8761) were added to each well and incubated for 16 hours. Culture medium was carefully removed from wells, and cells were cultured in RPMI-1640 medium supplemented with 10% FBS. After 48 hours, MNK28 and SGC7901 cells were cultured in RPMI-1640 medium containing 10% FBS and 10 mg/mL of neomycin (Sigma-Aldrich, CAS: 1404-04-2). MNK28 and SGC7901 cells were cultured in RPMI-1640 medium containing 10% FBS and 2 mg/mL of neomycin. The culture medium was changed, with neomycin added, every 3 days.

### Transwell migration assay

Matrigel was diluted 1:8 (BD Matrigel, 354234) and used to coat the upper chamber surface of the bottom membrane of the Transwell chamber (Corning, 3412). MNK28 and SGC7901 cells were cultured in RPMI-1640 medium without FBS. After 24 hours, MNK28 and SGC7901 cells were seeded (5 × 10^5^ cells/well) into a Transwell chamber for 24 and 48 hours, respectively. The Transwell chamber was removed, and the culture medium was discarded. The chamber was washed twice with calcium-free PBS, fixed with methanol for 30 minutes, and allowed to dry. Cells were stained with 0.1% crystal violet for 20 minutes, after which the upper nonmigrated cells were gently wiped off with a cotton swab, and remaining cells were washed three times with PBS. Cells were immediately observed in three fields under 100× magnification and then counted.

### miRNA-target interactions

miRWalk is an improved version of the previous database. It stores the predicted data obtained with a machine learning algorithm, including experimentally verified miRNA-target interactions [30].

### Database of immune cell expression

The Database of Immune Cell Expression (DICE; https://dice-database.org/landing) identified the expression quantitative trait loci for a total of 12,254 unique genes, which represent 61% of all protein-coding genes that are expressed across different cell types. Strikingly, a large fraction of these genes (41%) showed a strong *cis*-association with genotype only in a single cell type. DICE also showed that biologic sex is associated with major differences in immune cell gene expression in a highly cell-specific manner.

### TIMER database analysis

TIMER (https://cistrome.shinyapps.io/timer/) is a comprehensive resource for systematic analysis of immune infiltrates across different types of cancer. It infers the abundance of tumor-infiltrating immune cells from gene expression profiles. Across 32 cancer types, there are 10,897 samples in the TIMER database that are used to estimate immune infiltrates. Gene expression levels are displayed with log2 RSEM. Somatic copy number alterations (SCNAs) in the TIMER database are defined by GISTIC 2.0, including deep deletion (–2), arm-level deletion (–1), diploid/normal (0), arm-level gain (1), and high amplification (2). Box plots are presented to show the distributions of each immune subset at each copy number status in selected cancer types. The infiltration level for each SCNA category is compared with normal, using the two-sided Wilcoxon rank sum test.

### Overall survival

Overall survival (OS) was calculated using the Kaplan-Meier Plotter (http://www.kmplot.com), which evaluates the impact of 54,675 genes on survival time of cancer patients. A total of 10,188 cancer samples were analyzed using the Kaplan-Meier Plotter, including 1,648 ovarian, 4,142 breast, 1,065 gastric cancer, and 2,437 lung sample microarray expression profiles [5]. In this study, we analyzed the effect of miR-588, CXCL5, CXCL9, and CXCL10 (high vs. low expression) on OS of GC patients using a log-rank *P* value and a hazard ratio (HR) with 95% confidence intervals (CIs).

### Statistical analysis

The statistical analyses were performed using SPSS 19.0 software (IBM, Armonk, NY). Differences between groups were compared using student’s *t* test, and all data were presented as mean ± SD. *P* < 0.05 was considered statistically significant.

## Supplementary Material

Supplementary Figure 1

Supplementary Tables
