# Supplementary material for: miR-588 is a prognostic marker in gastric cancer
Source: Aging (Albany NY). 2020 Dec 15;13(2):2101–17. doi: 10.18632/aging.202212 (PMC7880400; doi:10.18632/aging.202212)
Supplement: Supplementary Figure 1 [file aging-13-202212-s001.pdf]

SUPPLEMENTARY FIGURE

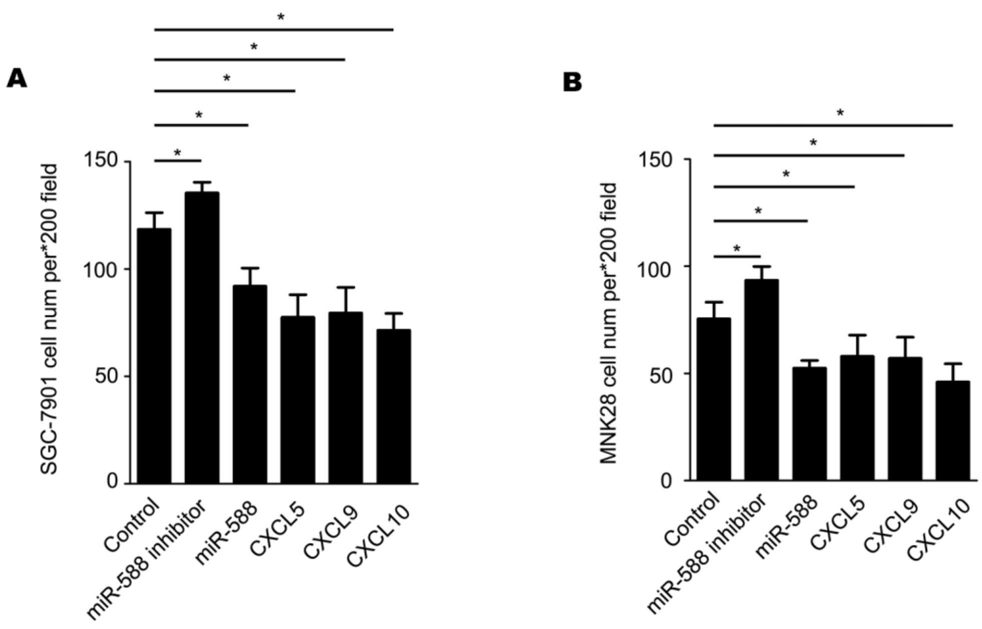

Supplementary Figure 1. Colony number statistics. (**A**, **B**) Cell number statistics of increased expression of miR-588, CXCL5, CXCL9, and CXCL10 in SGC-7901 and MNK28 cells. Each column represents the mean ± SD from three repeats. *P<0.05
