# Supplementary material for: miR-588 is a prognostic marker in gastric cancer
Source: Aging (Albany NY). 2020 Dec 15;13(2):2101–17. doi: 10.18632/aging.202212 (PMC7880400; doi:10.18632/aging.202212)
Supplement: Supplementary Tables [file aging-13-202212-s002.pdf]

SUPPLEMENTARY TABLES

Supplementary Table 1. Down expression.

logFC	AveExpr	t	P.Value	adj.P.Val	B	
hsa-miR-623	-1.09057	6.549142	-8.80011	1.67E-13	1.20E-10	20.38652
hsa-miR-508-3p	-1.96046	5.468348	-8.08192	4.53E-12	1.09E-09	17.18135
hsa-miR-588	-1.51453	4.875423	-7.85186	1.30E-11	2.33E-09	16.15907
hsa-miR-455-5p	-1.77742	3.987827	-7.64756	3.30E-11	3.39E-09	15.25431
hsa-miR-506	-2.1418	7.50375	-7.61231	3.87E-11	3.48E-09	15.09856
hsa-miR-127-3p	-1.57303	7.387447	-7.58293	4.43E-11	3.54E-09	14.96879
hsa-miR-26b*	-2.92071	4.143697	-7.5732	4.92E-11	3.54E-09	14.87005
hsa-miR-503	-2.16901	10.67893	-7.45041	8.08E-11	4.47E-09	14.38464
hsa-miR-30a*	-2.13099	10.9922	-7.38044	1.11E-10	5.32E-09	14.07696
hsa-miR-453	-1.71565	4.260195	-7.34927	1.28E-10	5.74E-09	13.94007
hsa-miR-601	-1.03993	6.687393	-7.31241	1.51E-10	6.39E-09	13.77831
hsa-miR-409-3p	-2.65592	5.426687	-7.28234	1.73E-10	6.60E-09	13.64648
hsa-miR-892b	-1.82718	10.63111	-7.26912	1.84E-10	6.60E-09	13.58856
hsa-miR-513a-3p	-2.46485	5.500691	-7.22644	2.23E-10	7.62E-09	13.4017
hsa-miR-186	-1.33433	4.564366	-7.14262	3.25E-10	1.06E-08	13.03538
hsa-miR-650	-1.07283	6.568176	-7.03796	5.20E-10	1.56E-08	12.57935
hsa-miR-216a	-2.58564	5.69957	-6.94177	8.00E-10	2.13E-08	12.16157
hsa-miR-30d	-1.59585	5.345926	-6.92725	8.54E-10	2.19E-08	12.09863
hsa-miR-325	-2.0648	4.616157	-6.82388	1.35E-09	3.04E-08	11.65153
hsa-miR-553	-1.53257	7.672097	-6.7866	1.60E-09	3.48E-08	11.49073
hsa-miR-551a	-1.12015	5.156939	-6.77253	1.70E-09	3.60E-08	11.43008
hsa-miR-675	-1.74128	10.80502	-6.65579	2.86E-09	5.14E-08	10.92835
hsa-miR-604	-2.03553	5.681132	-6.54177	4.73E-09	7.61E-08	10.44075
hsa-miR-221*	-3.09165	2.258321	-6.60084	5.73E-09	8.76E-08	10.30633
hsa-miR-222*	-1.15572	4.151699	-6.35285	1.08E-08	1.58E-07	9.638564
hsa-miR-1228*	-1.21224	6.563631	-6.34919	1.10E-08	1.58E-07	9.623115
hsa-miR-196b	-1.82046	11.12268	-6.33258	1.18E-08	1.67E-07	9.552928
hsa-miR-517a	-1.23927	6.780687	-6.28023	1.49E-08	1.94E-07	9.332261
hsa-miR-542-5p	-1.05102	5.969439	-6.18557	2.24E-08	2.74E-07	8.934774
hsa-miR-769-5p	-1.30587	6.19839	-6.15676	2.54E-08	3.05E-07	8.814213
hsa-miR-21	-1.31064	5.477165	-6.11344	3.07E-08	3.44E-07	8.633332
hsa-miR-491-5p	-1.32198	5.069569	-5.99578	5.09E-08	5.30E-07	8.144448
hsa-miR-517*	-1.10105	5.229021	-5.96219	5.87E-08	6.03E-07	8.005535
hsa-miR-509-3p	-1.17908	6.749489	-5.95705	6.01E-08	6.08E-07	7.984289
hsa-miR-144*	-1.31969	3.173495	-5.89589	7.80E-08	7.48E-07	7.732229
hsa-miR-452*	-1.09049	4.566291	-5.86796	8.78E-08	7.99E-07	7.617484
hsa-miR-31*	-1.00541	7.504884	-5.85594	9.24E-08	8.31E-07	7.568151
hsa-miR-17*	-2.28738	5.324781	-5.82977	1.06E-07	9.10E-07	7.438949
hsa-miR-889	-1.27726	6.011166	-5.78654	1.24E-07	1.00E-06	7.284147
hsa-miR-641	-1.17182	4.597678	-5.56242	3.18E-07	2.27E-06	6.37683
hsa-miR-136	-1.07475	5.742556	-5.54811	3.38E-07	2.36E-06	6.319435

hsa-miR-626	-1.66122	3.141684	-5.48325	4.42E-07	2.97E-06	6.060112
hsa-miR-616*	-1.10678	3.661107	-5.32404	8.53E-07	5.07E-06	5.42954
hsa-miR-568	-1.01959	4.705985	-5.27816	1.03E-06	6.01E-06	5.249448
hsa-miR-328	-1.16737	4.191332	-5.16799	1.61E-06	8.63E-06	4.820174
hsa-miR-627	-1.08764	6.456891	-5.13898	1.81E-06	9.49E-06	4.7079
hsa-miR-30c-1*	-1.07413	3.824865	-4.9727	3.52E-06	1.67E-05	4.070582
hsa-miR-422a	-1.05932	11.97417	-4.69587	1.04E-05	4.45E-05	3.034844
hsa-let-7b*	-1.35315	6.756759	-4.66151	1.19E-05	5.02E-05	2.908596
hsa-miR-577	-1.01248	6.601072	-4.50947	2.12E-05	8.07E-05	2.356545
hsa-miR-595	-1.0686	6.970834	-4.34129	3.98E-05	0.000137	1.758789
hsa-miR-888	-1.03496	3.918741	-4.25547	5.46E-05	0.00018	1.459191
hsa-miR-548d-5p	-1.21374	-0.2145	-2.5778	0.012949	0.02357	-3.32941

Supplementary Table 2. Up expression.

logFC	AveExpr	t	P.Value	adj.P.Val	B	
hsa-miR-99a	1.152462	5.258632	8.153983	3.25E-12	1.09E-09	17.5022
hsa-miR-944	1.406563	5.652367	7.773946	1.85E-11	2.57E-09	15.81363
hsa-miR-142-3p	1.283312	6.458629	7.741617	2.15E-11	2.57E-09	15.67043
hsa-miR-628-3p	1.526372	4.249315	7.500179	6.84E-11	4.47E-09	14.54973
hsa-miR-199a-3p	1.952701	9.107736	7.450222	8.09E-11	4.47E-09	14.38383
hsa-miR-550	1.005675	6.915845	7.117143	3.64E-10	1.14E-08	12.92424
hsa-miR-132	1.356283	6.808491	6.999866	6.17E-10	1.77E-08	12.41373
hsa-miR-129-5p	1.30892	7.086793	6.968717	7.09E-10	1.96E-08	12.27848
hsa-miR-455-3p	1.767636	8.84766	6.90826	9.29E-10	2.30E-08	12.01638
hsa-miR-768-3p_v11.0	1.673263	8.871763	6.887743	1.02E-09	2.44E-08	11.92757
hsa-miR-187	1.835561	5.045098	6.863398	1.19E-09	2.76E-08	11.78192
hsa-miR-205	1.018429	5.259305	6.738202	1.98E-09	3.95E-08	11.28231
hsa-miR-760	1.063167	4.859941	6.737875	1.99E-09	3.95E-08	11.2809
hsa-miR-1234	1.947934	9.551287	6.732652	2.03E-09	3.95E-08	11.25843
hsa-miR-372	1.1028	6.158762	6.699907	2.35E-09	4.33E-08	11.11769
hsa-miR-129*	1.761261	9.501502	6.540045	4.76E-09	7.61E-08	10.43339
hsa-miR-219-1-3p	1.009744	5.691691	6.535262	4.87E-09	7.61E-08	10.41299
hsa-miR-10a*	1.866967	9.511416	6.459305	6.80E-09	1.02E-07	10.08968
hsa-miR-218-2*	1.043867	4.052268	6.297851	1.38E-08	1.87E-07	9.406468
hsa-let-7d*	1.30925	5.41073	6.124393	2.92E-08	3.34E-07	8.679042
hsa-miR-296-5p	1.077886	6.445517	5.875889	8.49E-08	7.99E-07	7.650031
hsa-miR-549	1.424454	7.587812	5.832339	1.02E-07	8.96E-07	7.471418
hsa-miR-383	1.014069	5.731851	5.814298	1.10E-07	9.33E-07	7.397583
hsa-miR-377	1.293892	5.21971	5.787812	1.23E-07	1.00E-06	7.289353
hsa-miR-504	1.008071	7.159726	5.761861	1.38E-07	1.10E-06	7.183509
hsa-miR-542-3p	1.692321	4.628916	5.756239	1.41E-07	1.10E-06	7.160603
hsa-miR-767-5p	1.224601	6.425277	5.728546	1.59E-07	1.21E-06	7.04792
hsa-miR-493	1.294646	5.647842	5.707468	1.73E-07	1.31E-06	6.962302
hsa-miR-629*	1.151874	7.739722	5.694919	1.83E-07	1.37E-06	6.911395
hsa-miR-369-5p	1.440635	3.504745	5.65163	2.19E-07	1.61E-06	6.736149
hsa-miR-130b*	1.160195	7.012859	5.626336	2.44E-07	1.75E-06	6.634021
hsa-miR-106a*	1.539118	7.654099	5.419566	5.76E-07	3.69E-06	5.806832
hsa-miR-326	1.319038	6.914494	5.417691	5.80E-07	3.69E-06	5.799397
hsa-miR-24-1*	1.016383	6.166835	5.410885	5.97E-07	3.70E-06	5.772413
hsa-miR-31	1.255649	2.166106	5.355559	7.66E-07	4.67E-06	5.538449
hsa-miR-323-3p	1.063336	4.667848	5.241296	1.20E-06	6.82E-06	5.105311
hsa-miR-758	1.299205	8.159182	5.227575	1.26E-06	7.04E-06	5.051789
hsa-miR-523	1.006537	4.841865	5.224428	1.28E-06	7.08E-06	5.039526
hsa-miR-7-2*	1.467803	3.238004	5.152703	1.74E-06	9.29E-06	4.748298
hsa-miR-22*	1.311184	7.680631	4.934871	4.09E-06	1.88E-05	3.927151
hsa-miR-541	1.072879	2.5879	4.645014	1.28E-05	5.33E-05	2.841068
hsa-miR-550*	1.048043	7.126609	4.58089	1.62E-05	6.46E-05	2.61454
hsa-miR-448	1.19898	6.074344	4.563634	1.73E-05	6.82E-05	2.551986

hsa-miR-384	1.02845	3.17483	4.500897	2.19E-05	8.29E-05	2.325754
hsa-miR-610	1.201362	6.763803	4.467241	2.49E-05	9.32E-05	2.205161
hsa-miR-27b*	1.119525	1.796291	4.457214	2.72E-05	9.91E-05	2.147479
hsa-miR-145*	1.008986	7.176582	4.378614	3.47E-05	0.000122	1.890239
hsa-miR-374a*	1.042186	3.579827	4.305475	4.59E-05	0.000155	1.629225
hsa-miR-567	1.202223	4.261043	4.293175	4.76E-05	0.000159	1.590344
hsa-miR-195	1.182474	5.967	4.291625	4.78E-05	0.000159	1.584941
hsa-miR-877	1.178657	7.247811	3.875256	0.000212	0.000618	0.179076
hsa-miR-940	1.370201	6.917529	3.799314	0.000276	0.000778	-0.06687
hsa-miR-941	1.329929	4.235937	3.775977	0.000299	0.000833	-0.14177
hsa-miR-494	1.111857	6.198712	3.674789	0.000421	0.001147	-0.46275
hsa-miR-218-1*	1.08959	5.10152	3.626263	0.000496	0.00133	-0.61447
hsa-miR-220c	1.440638	2.012449	3.453163	0.000875	0.002201	-1.14369
hsa-miR-1226*	1.029277	6.380164	3.420158	0.000974	0.002415	-1.24242
hsa-miR-100	1.054828	7.212845	3.386523	0.001085	0.002662	-1.34231
hsa-miR-484	1.142778	6.841757	3.338582	0.001264	0.00298	-1.48339

Supplementary Table 3. Up expression.

miR-588 UP	5-TTGGCCACAATGGGTTAGAAC-3
miR-588 DOWN	5-CAGTGCAGGGTCCGAGGTAT-3
CXCL5 UP	5-TGTAAGTTCTGTGCTGCTGTG-3
CXCL5 DOWN	5-CTGCGGGATTTCTCTCTTGC-3
CXCL9 UP	5-ATGAGGATGAAAGTGGTGATTGG-3
CXCL9 DOWN	5-GGTGTTGGTGTTGAATAGAAAGC-3
CXCL10 UP	5-ATGAGGATGAAAGTGGTGATTGG-3
CXCL10 DOWN	5-GGTGTTGGTGTTGAATAGAAAGC-3
U6 UP	5-CTCGCTTCGGCAGCACATATACT-3
U6 DOWN	5-ACGCTTCACGAATTTGCGTGTC-3
GAPDH UP	5-GCGGGGCTCTCCAGAACATC-3
GAPDH DOWN	5-TCCACCACTGACACGTTGGC-3
